# Acute Flaccid Myelitis Among Persons Aged ≤21 Years — United States, August 1–November 13, 2014

**Published:** 2015-01-09

**Authors:** Division of Viral Diseases, National Centers for Immunization and Respiratory Diseases, CDC; Division of Vector-Borne Diseases, Division of High-Consequence Pathogens and Pathology, National Center for Emerging and Zoonotic Infectious Diseases, CDC; Children’s Hospital Colorado; Council of State and Territorial Epidemiologists

In August 2014, physicians at Children’s Hospital Colorado in Aurora, Colorado, noted a cluster of cases of acute limb weakness among children (1). Most patients were found to have distinctive abnormalities of the central spinal cord (i.e., gray matter) on magnetic resonance imaging, and most reported a respiratory or febrile illness preceding the onset of neurologic symptoms. On September 12, the Colorado Department of Public Health and Environment alerted CDC about this cluster. These cases coincided with a national outbreak of severe respiratory disease among children caused by enterovirus D68 (EV-D68) (2).

On September 26, CDC issued a health advisory requesting state and local health departments to report cases and send specimens to CDC for testing (3). A case was defined as acute onset of focal limb weakness occurring on or after August 1, 2014, and a magnetic resonance image showing a spinal cord lesion largely restricted to gray matter in a patient aged ≤21 years.

As of November 13, CDC had verified reports of 88 cases in 32 states (Figure). The median age of patients was 7.6 years (range = 5 months–20 years), and 54 (61%) were males. Limb weakness was asymmetrical in most patients. Cranial nerve motor dysfunction was reported in 30 (34%) cases. Six (7%) patients had altered mental status, and three (3%) had seizures. Most patients reported a respiratory illness (81%), a febrile illness (68%), or both, occurring before neurologic symptom onset; 8% had neither condition. Among 86 patients for whom past medical history was reported, 65 (76%) were previously healthy, and 21 (24%) had underlying illnesses, most commonly asthma (nine [10%]). All but one patient was hospitalized because of neurologic illness, and 17 (19%) required ventilator support. Among 80 patients from whom cerebrospinal fluid was obtained, 68 (85%) showed a moderate pleocytosis and normal or mildly elevated protein. Information regarding current clinical status was reported for 77 patients (median follow-up = 19 days). Of those, 49 (64%) reported some symptom improvement, and 28 (36%) showed no improvement; none were fully recovered. No deaths were reported.

Among 71 patients with cerebrospinal fluid testing performed by their health care providers, state and local public health departments, or CDC, no enteroviruses or other pathogens have been confirmed to date. Among 41 patients whose upper respiratory tract samples were available for enterovirus/rhinovirus testing at CDC, 17 (41%) tested positive: eight (20%) for EV-D68 and nine (22%) for eight other enterovirus/rhinovirus types. Of the 19 patients whose upper respiratory tract samples were obtained <14 days from respiratory illness onset, 10 (53%) were positive: seven (37%) for EV-D68 and three (16%) for rhinoviruses. Laboratory testing for other pathogens is ongoing.

On November 7, CDC published interim clinical management considerations, summarizing expert opinion based on current evidence on management and care of children with acute flaccid myelitis (4). CDC continues to collaborate with partners nationally to investigate reported cases, risk factors, and possible etiologies of this condition. Although the specific causes of this illness are still under investigation, and causal relationship to EV-D68 has not yet been substantiated, being up to date on all recommended vaccinations is essential to prevent a number of severe diseases. Vaccine-preventable diseases include poliomyelitis, which is caused by poliovirus; infection with this enterovirus can present with acute flaccid paralysis. There are also numerous other vaccine-preventable diseases that can result in severe illness. Prevention of viral infections includes general hygienic measures, such as frequent hand washing with soap and water, avoiding close contact with sick persons, and disinfecting frequently touched surfaces. Additional information is available at http://www.cdc.gov/flu/protect/habits/index.htm. If a child appears to have a sudden onset of weakness in arms or legs, caregivers should contact a health care provider to have the child assessed for possible neurologic illness. Health care providers are encouraged to report patients meeting the case definition to their state or local health department. Health departments should report patients with illness meeting the case definition to CDC using a brief patient summary form* and may contact CDC by e-mail to arrange further laboratory testing (limbweakness@cdc.gov). Additional information is available at http://www.cdc.gov/ncird/investigation/viral/sep2014.html.

## Figures and Tables

**FIGURE:**
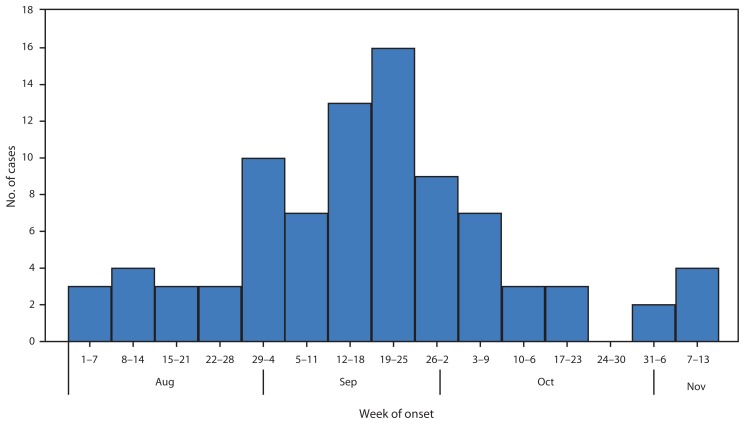
Number of confirmed cases of neurologic illness with limb weakness (N = 87), by week of onset — United States, August 1–November 13, 2014* * Exact onset date was not reported for one case (for this case the neurologic symptom onset was reported in an unspecified date during the last week of September).

## Footnotes

* Available at http://www.cdc.gov/ncird/downloads/patient-summary-form.pdf.
